# Hepatic encephalopathy and spontaneous bacterial peritonitis are associated with increased liver-related readmissions in cirrhosis

**DOI:** 10.3389/fmed.2025.1417222

**Published:** 2025-01-31

**Authors:** Shan Wang, Lin Zhang, Jin Li, Jiajun Feng, Jie Gao, Rui Huang

**Affiliations:** ^1^Peking University Hepatology Institute, Peking University People’s Hospital, Beijing, China; ^2^Department of Liver Diseases, The First Hospital of Lanzhou University, Lanzhou, China; ^3^The First Department of Neurology, The Third People’s Hospital of Liaoyang, Liaoyang, China; ^4^Department of Marketing, School of Business, Renmin University of China, Beijing, China; ^5^Department of Hepatobiliary Surgery, Peking University People’s Hospital, Beijing, China

**Keywords:** liver cirrhosis, readmission, hepatic encephalopathy, spontaneous bacterial peritonitis, hypertension

## Abstract

**Introduction:**

Liver disease remains a significant global health concern. In China, the number of patients with liver cirrhosis is estimated to reach 7 million. In addition to the high risk of death, cirrhosis leads to several severe complications. Patients with cirrhosis have significantly longer hospital stays and higher total hospital costs than those without cirrhosis. We aimed to investigate the predictors of readmission among patients with cirrhosis in China.

**Materials and methods:**

We conducted a retrospective study to evaluate adult patients with cirrhosis. Data on various sociodemographic, clinical, and hospitalization characteristics were collected. We defined the primary endpoint as the first liver-related readmission occurring within 30–90 days of initial hospitalization. Adult patients with cirrhosis admitted to our hospital between January 2009 and December 2022 were included. Differences between groups were analyzed using Student’s t-test and chi-square test. Logistic and multiple linear regression analyses were performed to identify predictors associated with readmission and the length of the first hospitalization.

**Results:**

In total, 1,285 patients were diagnosed with cirrhosis. Among these patients, 767 (59.7%) were males, and the mean age was 58.9 ± 12.3 years. Seventy-two (5.6%) and 154 (12.0%) patients were readmitted within 30 and 90 days, respectively. Compared with those who were not readmitted, patients readmitted at 30-day and 90-day had a higher proportion of males, ascites, spontaneous bacterial peritonitis, electrolyte abnormalities, higher Child-Pugh–Turcotte scores, longer initial hospital stays, and higher initial hospitalization costs. Logistic regression analysis indicated that hepatic encephalopathy, spontaneous bacterial peritonitis, diabetes, and ascites were predictors of 30- and 90-day readmission. Hypertension and spontaneous bacterial peritonitis were significant predictors of the length of the first hospitalization.

**Conclusion:**

Patients with cirrhosis presenting with hepatic encephalopathy, ascites, and spontaneous bacterial peritonitis may have a higher risk of rehospitalization.

## 1 Introduction

Liver disease remains a significant global health concern, resulting in approximately 2 million deaths annually worldwide, with 1 million fatalities attributed to cirrhosis complications. Cirrhosis is the 11th most common cause of death globally (1). In 2017, cirrhosis led to over 1.32 million deaths, comprising 2.4% of all global deaths (2). In China, the number of patients suffering from liver cirrhosis is estimated to approach 7 million (3), in whom approximately 460,000 new cases of hepatocellular carcinoma (HCC) are reported each year (4). Patients with compensated cirrhosis have a 4.7 times higher risk of death than the general population, whereas those with decompensated cirrhosis have a 9.7 times higher risk (5). Besides the high risk of death, cirrhosis leads to several severe complications, including ascites, variceal hemorrhage, hepatic encephalopathy (HE), HCC, spontaneous bacterial peritonitis (SBP), and malnutrition. These complications can significantly affect the patients’ quality of life, prevent them from working due to worsened physical and mental health, and impose substantial financial burdens (5, 6). Compared to individuals without cirrhosis, patients with cirrhosis experience significantly longer hospital stays and higher total hospital charges (7). Additionally, those with comorbid cirrhosis face a higher likelihood of 30-day and 90-day readmissions than patients without cirrhosis (7).

Patients with advanced liver disease have high readmission rates (8). Previous studies estimated the 30-day readmission rate in patients with cirrhosis to be 26% (9). Similarly, the readmission rate within 1 year was reported to be 28.7% in China (10). A study conducted in Texas showed that patients with chronic liver disease (25%) had a higher 30-day readmission rate than those with congestive heart failure (21.9%) or chronic obstructive pulmonary disease (20.6%) (11). Readmission of inpatients with cirrhosis is associated with a deteriorated quality of life and a higher risk of death (12). These readmissions not only pose challenges to patient health but also impose an increased financial burden (13). In the United States, the cost of cirrhosis-related hospitalizations doubled from $4.8 billion to $9.8 billion between 2001 and 2011 (14).

Numerous factors are associated with readmission in patients with cirrhosis, including socioeconomic characteristics, cirrhosis type, hepatorenal syndrome, ascites, variceal hemorrhage, HCC, Charlson Comorbidity Index, and HE (13, 15). In particular, HE has been identified as a significant predictor of 30-day and 90-day readmission in patients with cirrhosis, along with the presence of >3 cirrhotic complications (15). These structural changes have led to the pathogenesis of cirrhosis in recent years (16) The application of antivirals for hepatitis B virus (HBV) and hepatitis C virus (HCV), as well as large-scale neonatal HBV vaccination, has reduced the prevalence of cirrhosis related to viral hepatitis (14, 17, 18). To date, non-alcoholic steatohepatitis (NASH) and alcohol-related liver disease are the most common causes of liver cirrhosis (19–21). Therefore, further research is needed to identify the factors that influence readmitted hospital admissions in patients with cirrhosis, to treat patients, and to avoid the deterioration of these conditions early. We conducted a retrospective study to gain insight into the predictors influencing readmission in patients with cirrhosis in China.

## 2 Materials and methods

### 2.1 Data source

We conducted a retrospective study at a single center to evaluate adult patients with cirrhosis. The study period spanned from January 2009 to December 2022. We identified patients through our hospital’s electronic medical records using admission diagnoses of cirrhosis with the International Classification of Diseases, 10th revision, Clinical Modification (ICD-10-CM) codes K74.100 and K74.607. We excluded patients with incomplete information, those who were lost to follow-up, or those who died during the index hospitalization. The study was conducted in accordance with the ethical guidelines outlined in the 1975 Declaration of Helsinki (6th revision, 2008) and was approved by the Ethics Committee of Peking University People’s Hospital (No. 2023PHB214-001). Given the retrospective nature of the study, the requirement for informed consent was waived, and none of the enrolled patients were asked to provide informed consent.

### 2.2 Participants

Adult patients with cirrhosis who were admitted to our hospital between January 2009 and December 2022 were included in this study.

### 2.3 Main measures

We collected a comprehensive range of sociodemographic, clinical, and hospitalization characteristics, including sex, age, type of medical insurance (Chinese resident medical insurance and Chinese worker medical insurance), and baseline liver disease etiology (alcohol-related liver disease, viral hepatitis, or other causes, such as non-alcoholic steatohepatitis, drug-induced liver injury, or autoimmune liver disease). Comorbidities were documented, including hypertension (I10. ×00 × 002), diabetes mellitus (E14.900 × 001), and chronic kidney disease (CKD) (N18.900 × 005). We also recorded liver disease-related complications, such as HCC (C22.70D), HE (K72.90B), and ascites (R18. ×00), variceal bleeding (K92.201), SBP (K65.016), hepatic hydrothorax (J94.80D), and electrolyte disturbances (E87.800 × 011, E87.600, E87.500, E87.102, and E87.001). In addition, we documented the Model for End-Stage Liver Disease (MELD) and Child-Pugh–Turcotte scores at the time of the patient’s first discharge.

Alcoholic liver disease was diagnosed when alcohol consumption was ≥40 g/day in males and ≥ 20 g/day in females, with a history of alcohol intake spanning at least 5 years (22). Liver diseases other than alcoholic liver disease or viral hepatitis were identified based on admission and discharge diagnoses, with only the first occurrence of each diagnosis recorded. Laboratory data were retrieved from the hospital’s clinical data center.

The MELD score was calculated using parameters such as total bilirubin level, creatinine level, international normalized ratio (INR), and history of cholestatic liver disease. The Child-Pugh–Turcotte score was also calculated, and patients were categorized into three groups: A, good hepatic function; B, moderately impaired hepatic function; and C, advanced hepatic dysfunction, based on five clinical and laboratory criteria: serum bilirubin, serum albumin, ascites, neurological disorder, and clinical nutrition status.

### 2.4 Hospitalization characteristics

The recorded hospitalization characteristics included cirrhosis complications observed during the first admission and readmission. These complications included volume-related issues such as ascites (R18. × 00), hepatic hydrothorax (J94.80D), edema (R60.900), SBP (K65.016), variceal bleeding (K92.201), HCC (C22.70D), HE (K72.90B), liver function deterioration, infections, or other related factors.

Liver function deterioration was diagnosed based on at least one of the following criteria (23, 24): an increase in Child-Pugh score by 2 points or more from baseline; total bilirubin (TBil) levels ≥51 μmol/L or an increase in TBil levels from baseline of ≥34.2 μmol/L; and any increase in prothrombin time (PT) from baseline of ≥3 s. Infections were diagnosed according to international and local guidelines. Patients may have had multiple liver-related reasons for admission, and the major reasons for each admission were recorded. Furthermore, we collected data on the cost of the initial admission and the length of stay for the first hospitalization, calculated from admission to discharge.

### 2.5 Study outcomes

We defined the primary endpoint as the first liver-related readmission occurring within 30–90 days of initial hospitalization. To capture episodes of hospitalization at other hospitals, we contacted patients who had liver-related readmissions to any hospital since their first hospitalization. Liver-related readmission was defined as previously described. All other readmissions were excluded from the analysis. If a patient had more than one readmission within 30 days, only the first readmission was considered for the analysis. The secondary endpoint was the length of hospitalization at the time of admission.

We collected comprehensive information on readmissions by accessing the electronic medical records of our hospital, including medical histories, physical examinations, daily notes, laboratory results, and discharge summaries. To track hospitalizations at other facilities, we contacted patients with liver-related readmissions via telephone or conducted in-person interviews during subsequent visits to our outpatient clinic or inpatient department.

### 2.6 Statistical analysis

The normality of the data was evaluated using the Kolmogorov–Smirnov test. Mean ± standard deviation (SD) was used to present as continuous variables were normally distributed. Categorical variables are presented as numbers (%). Differences between groups were analyzed using Student’s t-test for variables such as age, MELD score, cost of admission, and length of stay. The chi-square test was used to analyze variables such as insurance, baseline liver disease, comorbidities, complications, treatment, and cause of readmission. Logistic regression analysis using a stepwise selection approach was performed to identify predictors associated with readmission, whereas multiple linear regression analysis was used to identify predictors associated with the length of the first hospitalization. For logistic model diagnostics, we employed the Hosmer-Lemeshow goodness-of-fit test to evaluate the calibration of our logistic regression models. A *p*-value <0.05 was considered statistically significant. All data analyses were conducted using SPSS version 23.0 (IBM Corp, Armonk, NY, USA).

## 3 Results

### 3.1 Baseline characteristics of patients with cirrhosis

We enrolled 1,285 patients who were admitted with cirrhosis. Their baseline characteristics are shown in Table 1. Within 30 days, 72 (5.6%) patients were readmitted, and within 90 days, the number increased to 154 (12.0%) patients. Among the patients, 767 (59.7%) were male, and the mean age was 58.9 ± 12.3 years. A total of 117 (9.1%) patients had no insurance coverage. Alcoholic cirrhosis was observed in 375 (29.2%) patients. On admission, 209 (16.3%), 1,192 (92.8%), and 88 (6.8%) patients presented with HE, ascites, and esophagogastric variceal bleeding, respectively. The mean MELD score was 9.9 ± 5.7. Regarding the Child-Pugh–Turcotte score, 726 (59.9%) and 219 (18.1%) patients were classified as Grade B and Grade C, respectively.

**Table 1:** Baseline characteristics of included patients.

Characteristics	All *n* = 1,285	30-day readmission			90-day readmission		
		Not readmitted *n* = 1,213	Readmitted *n* = 72	*p-*value	Not readmitted *n* = 1,131	Readmitted *n* = 154	*p-*value
Age (years), mean ± SD	58.9 ± 12.3	58.9 ± 12.4	59.2 ± 11.0	0.802	58.7 ± 12.5	60.3 ± 10.9	0.126
Male, *n* (%)	767 (59.7)	715 (58.9)	52 (72.2)	0.016	663 (58.6)	104 (67.5)	0.020
Insurance, *n* (%)				0.196			0.334
Any insurance	1,168 (90.9)	1,100 (90.7)	68 (94.4)		1,026 (90.7)	142 (92.2)	
No insurance	117 (9.1)	113 (9.3)	4 (5.6)		105 (9.3)	12 (7.8)	
Baseline liver disease, *n* (%)							
Alcohol	375 (29.2)	340 (28.0)	35 (48.6)	< 0.001	311 (27.5)	64 (41.6)	< 0.001
HBV infection	478 (37.2)	458 (37.8)	20 (27.8)	0.103	433 (38.3)	45 (29.2)	0.033
HCV infection	108 (8.4)	98 (8.1)	10 (13.9)	0.120	89 (7.9)	19 (12.3)	0.064
Other^a^	441 (34.3)	422 (34.8)	19 (26.4)	0.090	396 (35.0)	45 (29.2)	0.091
Co-morbidities, *n* (%)							
Hypertension	429 (33.4)	397 (32.7)	32 (44.4)	0.029	362 (32.0)	67 (43.5)	0.003
Diabetes	340 (26.5)	313 (25.8)	27 (37.5)	0.023	283 (25.0)	57 (37.0)	0.001
Chronic kidney disease	130 (10.1)	118 (9.7)	12 (16.7)	0.052	110 (9.7)	20 (13.0)	0.133
Complications, *n* (%)							
Hepatocellular carcinoma	235 (18.3)	220 (18.1)	15 (20.8)	0.329	200 (17.7)	35 (22.7)	0.082
Hepatic encephalopathy	209 (16.3)	182 (15.0)	27 (37.5)	< 0.001	158 (14.0)	51 (33.1)	< 0.001
Ascites	1,192 (92.8)	1,120 (92.3)	72 (100.0)	0.004	1,040 (92.0)	152 (98.7)	< 0.001
Variceal bleeding	88 (6.8)	83 (6.8)	5 (6.9)	0.558	77 (6.8)	11 (7.1)	0.491
Spontaneous bacterial peritonitis	197 (15.3)	168 (13.8)	29 (40.3)	< 0.001	148 (13.1)	49 (31.8)	< 0.001
Hepatic hydrothorax	116 (9.0)	113 (9.3)	3 (4.2)	0.341	109 (9.6)	7 (4.5)	0.253
Electrolyte abnormalities	603 (46.9)	577 (47.6)	26 (36.1)	< 0.001	542 (47.9)	61 (39.6)	< 0.001
MELD^b^ score, mean ± SD	9.9 ± 5.7	10.4 ± 5.7	9.6 ± 5.1	0.759	10.4 ± 5.8	9.7 ± 5.4	0.735
Child-Pugh-Turcotte Score, *n* (%)				< 0.001			< 0.001
A	273 (21.2)	268 (22.1)	5 (6.9)		263 (23.3)	10 (6.5)	
B	763 (59.4)	726 (59.9)	37 (51.4)		671 (59.3)	92 (59.7)	
C	249 (19.4)	219 (18.1)	30 (41.7)		197 (17.4)	52 (33.8)	

a Non-alcoholic steatohepatitis, drug-induced liver injury, autoimmune liver disease, etc.

b Model for end-stage liver disease.

HBV, hepatitis B virus; HCV, hepatitis C virus.

Considering 30-day and 90-day readmission cases This subgroup consisted of a higher proportion of males (30-day: 52 [72.2%] vs. 715 [58.9%], *p* = 0.016; 90-day: 104 [67.5%] vs. 663 [58.6%], *p* = 0.020) and individuals with alcoholic cirrhosis (30-day: 35 [48.6%] vs. 340 [28%], *p* < 0.001; 90-day: 64 [41.6%] vs. 311 [27.5%], *p* < 0.001) compared to those who did not experience readmission within the 30-day or 90-day timeframe. Patients admitted for 90 days had a lower proportion of HBV infections (45 [29.2%] vs. 433 [38.3%], *p* = 0.016). Furthermore, among the readmitted patients, a higher prevalence of HE (30-day: 27 [37.5%] vs. 182 [15.0%], *p* < 0.001; 90 day: 51 [33.1%] vs. 158 [14%], *p* < 0.001), ascites (30 day: 72 [100%] vs. 1,120 [92.3%], *p* = 0.004; 90-day: 152 [98.7%] vs. 1,040 [92.0%], *p* < 0.001), SBP (30-day: 29 [40.3%] vs. 168 [13.8%], *p* < 0.001; 90-day: 49 [31.8%] vs. 148 [13.1%], *p* < 0.001), and electrolyte abnormalities (30-day: 26 [36.1%] vs. 577 [47.6%], *p* < 0.001; 90-day:61 [39.6%] vs. 542 [47.9%], *p* < 0.001) was observed. Notably, readmitted patients had significantly higher Child-Pugh Turcotte scores than those who were not readmitted (30-day, *p* < 0.001, 90-day, *p* < 0.001).

### 3.2 Hospitalization characteristics

The major causes of first admission were volume-related (568, 44.2%), liver function deterioration (285, 22.2%), HE (101, 7.9%), and other causes (164, 12.8%). Among the patients readmitted within 30 and 90 days, a higher proportion had volume-related issues, and HE was the primary reason for their initial hospitalization. The mean cost for first admission was 25,035 ± 51,529 Renminbi (RMB). Among the patients readmitted within 30 and 90 days, there was a higher prevalence of longer initial hospital stays (30-day: 19.8 ± 13.1 vs. 15.1 ± 12.0 days, *p* = 0.001; 90-day: 17.8 ± 11.5 vs. 15.0 ± 12.2 days, *p* = 0.007) and higher initial hospitalization costs (30-day: 33833 ± 29,829 vs. 24,509 ± 52,504 RMB, *p* = 0.136; 90-day: 27461 ± 26,636 vs. 24,702 ± 54,056 RMB, *p* = 0.533). The primary causes of readmission within 30 days were volume-related issues (*n* = 30, 41.7%), liver function deterioration (*n* = 12, 16.7%), and HE (*n* = 11, 15.3%). For readmissions within 90 days, the major causes were volume-related issues (*n* = 80, 51.9%), deterioration of liver function (*n* = 20, 12.9%), and other miscellaneous causes (*n* = 15, 9.7%). Of the patients who were readmitted within 30 days, 33 (45.8%) were readmitted for the same cause. Similarly, among patients readmitted within 90 days, 72 (46.8%) were readmitted for the same cause (Table 2).

**Table 2:** Hospitalization characteristics.

Index hospitalization	All *n* = 1,285	30-days readmission			90-days readmission		
		Not readmitted *n* = 1,213	Readmitted *n* = 72	*p*-value	Not readmitted *n* = 1,131	Readmitted *n* = 154	*p*-value
Major cause for the first admission, *n* (%)				0.011			< 0.001
Volume-related	568 (44.2)	530 (43.7)	38 (52.8)	(--)	485 (42.9)	83 (53.9)	(--)
Variceal bleeding	46 (3.6)	46 (3.8)	0 (0)	(--)	42 (3.7)	4 (2.6)	(--)
Hepatocellular carcinoma	58 (4.5)	53 (4.4)	5 (6.9)	(--)	48 (4.2)	10 (6.5)	(--)
Hepatic encephalopathy	101 (7.9)	90 (7.4)	11 (15.3)	(--)	82 (7.3)	19 (12.3)	(--)
Liver function deterioration	285 (22.2)	272 (22.4)	13 (18.1)	(--)	261 (23.1)	24 (15.6)	(--)
Infection	63 (4.9)	63 (5.2)	0 (0)	(--)	63 (5.6)	0 (0)	(--)
Other	164 (12.8)	159 (13.1)	5 (6.9)	(--)	150 (13.3)	14 (9.1)	(--)
Cost for the first admission (RMB^a^), mean ± SD	25,035 ± 51,529	24,509 ± 52,504	33,833 ± 29,829	0.136	24,702 ± 54,056	27,461 ± 26,636	0.533
Length of stay (days) mean ± SD	15.3 ± 12.1	15.1 ± 12.0	19.8 ± 13.1	0.001	15.0 ± 12.2	17.8 ± 11.5	0.007
Major cause for readmission, *n* (%)							
Volume-related^b^	(--)	(--)	30 (41.7)	(--)	(--)	80 (51.9)	(--)
Variceal bleeding	(--)	(--)	3 (4.2)	(--)	(--)	5 (3.2)	(--)
Hepatocellular carcinoma	(--)	(--)	3 (4.2)	(--)	(--)	8 (5.2)	(--)
Hepatic encephalopathy	(--)	(--)	11 (15.3)	(--)	(--)	13 (8.4)	(--)
Deterioration of liver function^c^	(--)	(--)	12 (16.7)	(--)	(--)	20 (12.9)	(--)
Infection	-	-	6 (8.3)	-	-	9 (5.8)	-
Other	-	-	7 (9.7)	-	-	15 (9.7)	-
Readmission for the same cause, *n* (%)	-	-	33 (45.8)	-	-	72 (46.8)	-

^a^ Renminbi. ^b^ ascites, hepatic hydrothorax, edema, or spontaneous bacterial peritonitis. ^c^ (1) an increase in Child-Pugh score from pre-visit of 2 points or more; (2) total bilirubin (TBil) ≥51 μmol/L or an increase in TBil from pre-visit of ≥34.2 μmol/L; (3) any increase in prothrombin time from pre-visit of ≥3S.

### 3.3 Predictors of 30-day and 90-day readmission

The results of the logistic regression analysis for the predictors of readmission within 30 and 90 days are presented in Table 3. Significant predictors of readmission within 30 days included HE and SBP. For readmission within 90 days, the significant predictors included diabetes, HE, ascites, and SBP (Table 3).

**Table 3:** Predictors of 30-day and 90-day readmission in the logistic regression analysis.

Characteristics	*β*	95% CI of *β*	*p*-value	OR
30-day readmission				
Hepatic encephalopathy	0.827	0.139–1.515	0.018	2.36
Spontaneous bacterial peritonitis	1.255	0.611–1.899	0.000	3.82
90-day readmission				
Diabetes	0.608	0.191–1.025	0.004	2.86
Hepatic encephalopathy	0.641	0.149–1.132	0.011	2.55
Ascites	1.662	0.203–3.121	0.025	2.23
Spontaneous bacterial peritonitis	0.808	0.338–1.279	0.001	3.37

### 3.4 Predictors of length of hospitalization at the first admission

Table 4 demonstrates that hypertension (*p* = 0.001) and SBP (*p* < 0.001) were significant predictors of the length of hospitalization of patients during their initial admission.

**Table 4:** Predictors of length of hospitalization at the first admission.

Characteristics	B^a^	SE	*β* estimate	*p-*value	95.0%CI for *β*
Males	0.627	0.696	0.90	0.367	−0.737–1.9925
Age (years)	−0.040	0.026	−1.55	0.122	−0.092–0.011
Baseline liver disease					
Alcohol	−0.764	0.761	−1.00	0.315	−2.257–0.728
Viral	0.320	0.647	0.49	0.621	−0.949–1.589
Co-morbidities					
Hypertension	1.781	0.642	2.77	0.006	0.521–3.042
Diabetes	−0.722	0.662	−1.09	0.276	−2.021–0.577
Chronic kidney disease	0.909	0.724	0.94	0.350	−0.998–2.817
Complications					
Hepatocellular carcinoma	0.124	0.787	0.16	0.875	−1.420–1.669
Hepatic encephalopathy	0.401	0.782	0.51	0.608	−1.134–1.936
Ascites	0.623	1.127	0.55	0581	−1.589–2.8347
Variceal bleeding	1.167	1.114	1.05	0.295	−1.019–3.353
Spontaneous bacterial peritonitis	3.572	0.806	4.43	0.000	1.991–5.154
Hepatic hydrothorax	0.137	0.994	0.14	0.890	−1.813–2.087
Electrolyte abnormalities	0.359	0.600	0.60	0.550	−0.819–1.536
Acute kidney injury	−4.010	4.403	−0.91	0.363	−12.648–4.628
MELD^a^ score	0.017	0.594	0.28	0.781	−0.100–0.133
Child-Pugh-Turcotte score					
B	−0.365	0.672	−0.54	0.587	−1.684–0.953
C	−0.152	1.056	0.14	0.886	−1.920–2.224
Constant	13.264	1.997	6.64	0.000	9.345–17.182

a Model for end-stage liver disease.

HCC, hepatocellular carcinoma; HCV, hepatitis C virus; HBV, hepatitis B virus; NAFLD, non-alcoholic fatty liver disease; NASH, non-alcoholic steatohepatitis; HE, hepatic encephalopathy; SBP, spontaneous bacterial peritonitis; CKD, chronic kidney disease; MELD: End-Stage Liver Disease; INR, international normalized ratio; EGVB, esophagogastric variceal bleeding; TBil, total bilirubin; PT, prothrombin time; RMB: Renminbi; T2DM, type 2 diabetes mellitus.

## 4 Discussion

Data and evidence on the rates and reasons for readmission among patients with cirrhosis in China are currently lacking. Moreover, the causes of cirrhosis in China differ significantly from those in Europe and the United States, with a higher proportion of cirrhosis cases attributed to viral hepatitis. Our study, which included a large sample size, identified the predictors of readmission in Chinese patients with cirrhosis. These findings provide valuable insights for the targeted management of complications, with the aim of reducing readmission risks and optimizing medical resource utilization for patients with cirrhosis in China. This retrospective study revealed that patients with cirrhosis who presented with HE, ascites, or SBP had a higher risk of rehospitalization. Additionally, the study found that hypertension and SBP could predict the length of hospitalization during initial admission. Along with variceal hemorrhage, ascites and HE are manifestations of hepatic decompensation in patients (25). Based on these results, it is crucial to focus on controlling the progression of patient complications, particularly HE, ascites, and SBP, in order to reduce readmissions in patients with cirrhosis and alleviate their financial burden. Diuretics, antibiotics, and albumin should be administered early and reasonably to control the progression of ascites and SBP. To control the progression of HE, the American Association for the Study of Liver Diseases recommends the assessment of covert HE in patients with cirrhosis, as covert HE is a precursor of overt HE (26). A prospective trial suggested that a high dose/frequency treatment of overt HE would reduce readmission within 30 days in patients with cirrhosis (27). By implementing these strict measures to manage the development of complications in patients with cirrhosis, we can improve their physical condition and overall quality of life, as well as reduce their readmission.

HE is a significant complication of decompensated cirrhosis and is associated with poor outcomes (28). Overt HE results in hospital admissions and high 1-year and 5-year mortality (29). The interplay among increased ammonia concentrations, alterations in amino acid metabolism, and inflammation is central to this disease (30). The presence of HE in patients with cirrhosis is thought to be associated with hospital readmission, increased mortality, impaired quality of life, and greater fall risk (31). A study concluded that HE was most strongly associated with readmission within 30 (OR 3.23, 95%CI 2.97–3.52) and 90 days (OR 3.07, 95%CI 2.86–3.30) (15). Patients who were diagnosed with HE had median survivals of 0.95 and 2.5 years for those aged <65 or ≥ 65 years, respectively (32). A cohort study of 1,560 patients concluded that patients with grade 3–4 HE during hospitalization had higher MELD scores and mortality (72%) than those with grade 1–2 or no HE (33). HE was also the main reason for repeated readmission within 3 months (34). Our study aligns with these findings, emphasizing the importance of early identification and intervention in controlling the progression. According to the practice guidelines, controlling precipitating factors of overt HE is of paramount importance. Special drug treatments, such as lactulose, antibiotics, and rifaximin, are part of management (26). This proactive approach serves as a robust strategy to effectively reduce readmissions in patients with cirrhosis.

Ascites and SBP are significant complications commonly observed in patients with cirrhosis and are closely linked to their worsening condition. Specifically, ascites has been associated with a higher risk of 30-day readmission (15). Other features of cirrhosis, including gastrointestinal bleeding, infections, and renal failure, pose significant risks to the patients’ overall outcomes. These factors contribute to higher readmission rates, and a consistent relationship exists between readmission and subsequent mortality (9). SBP is a complication that specifically occurs in patients with ascites and tends to recur (35). Reports have indicated that the 30-day readmission rate for SBP is 25.6% (36). Ascites is a common complication of decompensated cirrhosis and increases the risk of further complications such as SBP and umbilical hernias, which may lead to readmission for further therapy (37). Our study concluded that ascites and SBP were associated with rehospitalization in patients with cirrhosis, which is similar to the reports above. Consequently, when patients develop ascites, it is essential to implement preventive measures against infections to mitigate the risks associated with SBP and reduce the likelihood of readmission. In this study, esophageal variceal bleeding did not increase the risk of readmission. This may be due to variceal eradication through early endoscopic control after endoscopic band ligation, which has been identified as a significant predictor of 90-day readmission in patients with cirrhosis. Similarly, a comprehensive review concluded that cirrhotic patients with diabetes had a higher risk of HE, elevated portal pressure, gastrointestinal bleeding, and increased susceptibility to bacterial infections and HCC (38). These factors contributed to the patient readmission rates. In a retrospective cohort study involving 12,442 patients who underwent liver transplantation, recipients with diabetes had longer hospital stays, higher peri-transplant mortality, inferior graft outcomes, and lower patient survival (39). Additionally, another study highlighted that patients with liver cirrhosis and type 2 diabetes mellitus (T2DM) had a significantly increased median length of hospital stay, doubled rate of non-cirrhosis-related admissions, and 1.35-fold increased rate of cirrhosis-related admissions compared to those without T2DM (40). While our study did not find diabetes to be a predictor of length of hospitalization, it revealed that diabetes affects long-term readmission in patients with cirrhosis. These findings suggest that patients with cirrhosis who also had diabetes were at a higher risk of prolonged hospital stay, and their overall condition would deteriorate if diabetes remained uncontrolled. Therefore, it is crucial to rigorously manage the development of comorbidities in patients with cirrhosis and diabetes to delay disease progression and improve outcomes.

In our cohort, we observed that hypertension and SBP were predictors of the length of hospitalization in patients with cirrhosis. Hypertension is a prevalent chronic disease worldwide and is often associated with other conditions, such as cardiovascular disease and non-alcoholic fatty liver disease (41, 42). Recent research has revealed several factors related to the length of hospital stay in patients with cirrhosis, including malnutrition, diabetes, infection, paracentesis use, SBP, and bariatric surgery (38, 43–46). Interestingly, there is limited research specifically exploring the relationship between hypertension and length of hospitalization in patients with cirrhosis. According to the literature, patients with cirrhosis often experience decreased splanchnic resistance and increased peripheral resistance. These changes are influenced by altered circulating vasoactive substances and the reactivity of the vasomotor and regulatory systems. Consequently, patients may present with hypotension, normotension, or hypertension (47, 48). One possible reason for the scarcity of studies is that arterial hypertension is not commonly observed in patients with cirrhosis. Some studies have suggested that hypertension exerts a protective effect against the occurrence of ascites or other complications related to circulatory dysfunction (49). Nevertheless, certain types of liver diseases, such as alcoholic fatty liver and HBV with renal involvement, may be accompanied by arterial hypertension (47). Our study population consisted predominantly of patients with alcohol-related and viral liver diseases, accounting for 74.3% of enrolled patients. This could explain why hypertension emerged as a significant predictor of the length of hospitalization in our study. Combined with previous literature, we believe that the prolonged hospital stay of patients with cirrhosis and hypertension may be related to kidney injury caused by hypertension and may also be related to the damage of some target organs by inflammatory factors released by the body during hypertension (48). However, further investigations are necessary to validate and elucidate the relationship between hypertension and length of hospitalization in patients with cirrhosis, particularly to explore its association with different types of liver diseases, such as alcohol-related and viral liver diseases. A potential research direction may be that hypertension leads to abnormal vasomotor function, which leads to abnormal secretion of hormones regulating blood pressure, adversely affecting liver function, and thus prolonging hospital stay in patients with cirrhosis.

Our study has several limitations. First, the indications for readmission varied over time, even within the same hospital, which was a major limitation of this retrospective analysis. Second, a notable omission in our study was the lack of data on specific treatments received by the patients, which could potentially serve as predictors of readmission and duration of hospitalization. Future studies should incorporate treatment data to provide more comprehensive insight. Third, the underlying mechanisms by which HE and SBP affect readmission rates in patients with cirrhosis have not yet been explored. Further investigations into these mechanisms are necessary as they could help develop more effective strategies to reduce readmission rates. Mortality is a key factor that may have influenced the outcomes of this study. However, because this was a retrospective study, we were unable to obtain mortality data, which is another limitation. Post-discharge management, including treatment of the underlying liver disease, medications for complications (such as beta-blockers, rifaximin, and diuretics), treatment of comorbidities, and patient adherence, could significantly affect the risk of readmission. Unfortunately, the lack of comprehensive post-discharge data prevented us from investigating this aspect. Moreover, non-liver-related deaths and readmissions are common among patients with cirrhosis. This area requires further exploration in future studies. Finally, although our study identified hypertension as a predictor of the length of hospitalization in patients with cirrhosis, we did not extensively explore this relationship. Future research should thoroughly examine the association between hypertension and hospitalization duration, particularly in the context of different liver diseases, such as alcohol-related and viral liver diseases. Our study highlighted hypertension as a predictor of the length of hospitalization in patients with cirrhosis; however, we did not extensively investigate this relationship. Therefore, additional research should be conducted to thoroughly explore the association between hypertension and the length of hospitalization, particularly considering different types of liver diseases, such as alcohol-related and viral liver diseases.

## 5 Conclusion

Patients with cirrhosis and conditions such as HE, ascites, SBP, and diabetes may have a higher risk of rehospitalization. Additionally, hypertension and SBP appear to be associated with the length of hospitalization in these patients. Clinicians might benefit from focusing on the early management of complications such as HE, SBP, and diabetes in patients with cirrhosis. However, these conclusions should be interpreted with caution, and additional studies are required to confirm these associations.

## Data Availability

The original contributions presented in the study are included in the article/supplementary material, further inquiries can be directed to the corresponding authors.
